# 1147. A study of high-dose influenza vaccination responses compared to standard-dose in lupus patients: an open- labeled, randomized controlled study

**DOI:** 10.1093/ofid/ofad500.988

**Published:** 2023-11-27

**Authors:** Sasicha Yingyounyong, Pintip Ngamjanyaporn, Prapaporn Pisitkun, Kobporn Boonnak, Thanitta Suangtamai, Supranee Thongpradit, Porpon Rotjanapan

**Affiliations:** Faculty of Medicine Ramathibodi Hospital, Mahidol University, Bangkok, Krung Thep, Thailand; Faculty of Medicine Ramathibodi Hospital, Mahidol University, Bangkok, Krung Thep, Thailand; Faculty of Medicine Ramathibodi Hospital, Mahidol University, Bangkok, Krung Thep, Thailand; Faculty of Medicine Siriraj Hospital, Mahidol University, Bangkok, Krung Thep, Thailand; Faculty of Medicine Ramathibodi Hospital, Mahidol University, Bangkok, Krung Thep, Thailand; Faculty of Medicine Ramathibodi Hospital, Mahidol University, Bangkok, Krung Thep, Thailand; Faculty of Medicine Ramathibodi Hospital, Mahidol University, Bangkok, Krung Thep, Thailand

## Abstract

**Background:**

Annual influenza vaccination at standard dosing in the general population is suggested. However, there may be more suitable for lupus patients.

**Objectives:**

To assess the immune responses among lupus patients receiving high dose (HD) and standard (SD) quadrivalent inactivated influenza vaccine, evaluate the adverse events of the HD, and monitor the incidence of influenza infection.

**Methods:**

A randomized parallel design, controlled trial was conducted from March 2021 to May 2022 at Ramathibodi Hospital. All lupus patients were stratified into two groups depending on the depth of immunosuppressive therapy received and randomized to receive either HD or SD in a 1:1 ratio. Patient demographics data and relevant information were retrieved. Blood samples were obtained for hemagglutination inhibition assay (HAI) before vaccination and 4-12 weeks after completion of the vaccination series to determine seroprotection and seroconversion rates.

**Results:**

One hundred twenty-eight lupus patients were enrolled, and 109 individuals completed the HAI analysis. 54/109 patients were in the high- (HI) with mean age (mean ± SD) of 34.3 (25.2-43.4) years, and 55/109 were in the low-immunosuppressive (LI) groups with mean age (mean ± SD) of 38.2 (30.2-46.2) (*p*=0.018). The majority of patients had a SLEDAI-2K score of 4. The seroprotection rates against H1N1, H3N2/Hongkong, H3N2/Cambodia, B/Victoria, and B/Yamagata strains in HI and LI groups at baseline after the first and second injections were outlined as follows 1.) HI; 74.1%, 82.1%, 76.9%, 75.9%, 79.6%; 85.2%, 89.3%, 88.5%, 85.2%, 88.9%; 89.5%, 89.3%, 88.5%, 93.9%, 88.9%, and 2.) LI; 76.4%, 84.6%, 93.1%, 74.5%, 89.1%; 92.7%, 100%, 100%, 94.5%, 96.4%; 92.7%, 100%, 100%, 94.5%, 94.5%. There was one documented influenza infection during the 9-month follow-up period. The patient was in the LI group and received the SD vaccine. 19/109 (17.4%) patients had grade 1 adverse events with no difference between SD and HD.

**Conclusion:**

Lupus patients’ immune response, especially those taking HI, may not be similar to healthy individuals. A higher dosage of influenza vaccination may provide a better seroprotection rate. However, real-world clinical effectiveness is yet to demonstrate, and further studies are required.

**Disclosures:**

**All Authors**: No reported disclosures

